# Performance and Welfare of Sows Exposed to Auditory Environmental Enrichment in Mixed or Collective Housing Systems

**DOI:** 10.3390/ani13071226

**Published:** 2023-03-31

**Authors:** Janaina Palermo Mendes, Fabiana Ribeiro Caldara, Maria Fernanda de Castro Burbarelli, Jean Kaique Valentim, Daniela Ferreira de Brito Mandú, Rodrigo Garófallo Garcia, Ibiara Correia de Lima Almeida-Paz, Agnes Markiy Odakura, Marconi Italo Lourenço da Silva

**Affiliations:** 1 Animal Science Postgraduate Program, Agricultural Sciences, Federal University of Grande Dourados (UFGD), Dourados 79804-970, MS, Brazil; 2 Department of Animal Production, School of Veterinary Medicine and Animal Science (FMVZ), São Paulo State University (UNESP), Botucatu 18610-034, SP, Brazil

**Keywords:** auditory stimulus, animal welfare, environmental enrichment, infrared thermography, reproductive performance, swine

## Abstract

**Simple Summary:**

Auditory environmental enrichment may be used to relieve animal stress and has been reported to have positive behavioral effects in pigs, considering it aims to improve restrictive living conditions associated with intensive production systems. This research aimed to assess the effects of auditory environmental enrichment on sows in mixed housing (caged until 35 days after insemination and then collective pens) or collective housing (caged until 72 h after insemination and then collective pens). The collective reproductive management did not demonstrate losses in the productive performance of swine, which may be an interesting management alternative as it reduces the time spent in individual facilities, improving the welfare of sows. These findings may be used to confront conservative protocols that indicate log periods in individual facilities to prevent gestational loses. The auditory environment enrichment also had positive influences on sows’ welfare, which was reflected in the performance of piglets; calm sows had heavier weaned litters, which may be considered another interesting finding for swine production.

**Abstract:**

The research aimed to assess the effects of auditory environmental enrichment on sows in mixed housing (caged until 35 days after insemination and then collective pens) or collective housing (caged until 72 h after insemination and then collective pens). Reproductive performance, body surface and eye temperature (ET) were evaluated as sows’ welfare indicators. A sample of 56 sows between 2nd and 6th parity was submitted to the treatments from artificial insemination to weaning. The sows were assigned in a randomized block design with a 2 × 2 factorial scheme of treatments: mixed housing—control (MH-C), collective housing—control (CH-C), mixed housing—music (MH-M) and collective housing—music (CH-M). Auditory enrichment consisted of exposing sows daily throughout gestation and lactation to 6 h of classical music divided into 2 h periods. The ET of pregnant sows in collective housing was lower than that of sows in mixed housing (33.77 °C vs. 34.91 °C). Pregnant and lactating sows exposed to auditory environmental enrichment exhibited lower ET compared to those that had no access to the stimulus (pregnant 33.36 °C vs. 34.32 °C and lactating 34.21 °C vs. 34.83 °C). No housing type effect was found on the reproductive performance parameter; however, piglets from sows submitted to auditory environmental enrichment, regardless of the type of housing during gestation, were heavier at weaning (6.32 kg vs. 5.57 kg). Collective or mixed housing does not affect sows’ reproductive performance; perhaps, auditory environmental enrichment reduced stress in the gestation and lactation phases and provided greater piglet weight gain at weaning.

## 1. Introduction

The worldwide increase in demand for meat products and the increasingly competitive market has directed industries to reach greater productivity, higher meat quality [1,2] and lower costs. Therefore, facilities have become smaller, aiming to house the largest number of animals in the smallest space [3]. Sows have been housed in individual pens during gestation and lactation to optimize space and increase reproductive control [4]. However, this system directly impacts sows’ welfare, since in crates, movements are restricted and the expression of natural species behaviors are significantly restricted, which causes behavioral issues, such as stereotypies and decreased maternal ability, especially in nest building before farrowing [5,6,7].

With global pressure regarding the welfare of production animals, alternatives to those systems, particularly concerning gestation crates, have been gradually replacing the individual model. Currently, the most widely accepted management for the gestation phase allows pregnant sows to be housed in individual pens for up to 35 days after mating, after which they are transferred to collective pens [8]. The reason for the adoption of this practice is that the stress of regrouping unfamiliar sows with each other prior to embryo nidation, which occurs around 17 to 24 days after fertilization, may lead to higher embryonic mortality rates [9].

With the aim to improve the quality of life for pregnant sows, recently, group housing systems have been proposed, in which females are inseminated in individual crates and transferred to collective pens after a few days. However, making it viable requires ways of reducing the stress of regrouping, establishment of social hierarchy and competition for food [4,10]. In this context, environmental enrichment may be a fundamental tool in controlling stress, considering it aims to improve restrictive living conditions associated with intensive production systems and to provide an opportunity for the expression of typical behaviors of the species, thus helping manage undesirable and harmful behaviors, such as aggressiveness, tail biting, sham chewing, belly nosing, etc. [11].

Auditory environmental enrichment may be used to relieve animal stress and has been reported to have positive behavioral effects in pigs and other species [12,13]. In addition, it may also break the silence, transforming an auditory monotonous environment into a more pleasant and productive one, which positively affects the cognitive development of animals [14]. For pregnant sows, Silva et al., [15] concluded that classical music promoted greater relaxation and reduction of agonistic behaviors and stereotypies, in addition to modulating physiological parameters that are indicators of stress, such as reduced respiratory rate. However, few reports were found relating the effects of auditory environmental enrichment on production parameters, as well as the evaluation of its benefits for other categories, such as lactating sows and their litters. Thus, the effects of environmental sound enrichment on aspects of welfare and productivity in swine production need to be better elucidated scientifically.

In the face of this, the present research was carried out aiming to assess the effect of auditory environmental enrichment on the performance and welfare of sows in mixed or collective housing during the gestation phase.

## 2. Material and Methods

All procedures of this study were approved by the Ethics Committee on the Use of Animals (CEUA) of the Federal University of Grande Dourados—UFGD, Dourados, Mato Grosso do Sul State, Brazil, under protocol n° 02/2020

### 2.1. Site Description

The experiment was conducted between February and June 2020 in a commercial piglet production unit hosting 3,500 sows in Ivinhema, Mato Grosso do Sul, Brazil. The site is located at 22°21′45″ S, 53°52′49″ W, and 406 m altitude. The climate in the region, according to the Köppen classification, is Aw, i.e., tropical climate with dry winters, with an average of 1200 to 1800 mm of annual rainfall and a mean annual temperature of 25 °C, possibly reaching highs up to 40 °C in spring and lows of 10 °C in winter.

During the experimental period, the ambient temperature was measured using thermo-hygrometers (model Novo Test TH802A^®^, NovotestBR, Vinhedo, SP, Brazil), positioned in the center of the pens and cages. Ambient temperature over the experimental period varied between 13.2 °C and 34.9 °C in the morning and 22.8 °C to 36.0 °C in the afternoon.

### 2.2. Animals, Experimental Design and Treatments

For the trial, 56 DanBred sows between the 2nd and 6th parturition were selected after weaning. Immediately after weaning, they were transferred to two identical gestation barns, 15 m from each other. The crates had 0.64 × 2.20 m and a compacted floor. All facilities were separated by enough physical space to ensure acoustic isolation between treatments, ensuring the absence of treatment bias. All pens and crates had the same environmental condition, and all management was the same in every treatment (except for the music).

There were 28 sows in each barn, according to the group of treatment (music or control), housed in individual cages. The experimental period began at the moment the sows were artificially inseminated and lasted until the end of the subsequent lactation phase, totaling 135 days (114 days of gestation and 21 days of lactation).

Sows of the collective housing treatment were transferred to group pens 72 h after artificial insemination (AI), while those in the mixed management group remained in individual cages until 35 days after insemination, and then they were transferred to collective pens. The stocking rate in the collective pens was 2.25 m^2^ per sow. The sows were assigned in a 2 × 2 factorial randomized block design, according to parturition order, to the following treatments (Table 1).

Approximately seven days prior to the expected delivery date, all sows were transferred to two identical farrowing rooms, in the respective barn, and housed in conventional farrowing crates with fully slatted floors and creep. The sows were fed with commercial corn–soybean meal-based diets, formulated to meet the nutritional requirements of each phase [16] (gestation AMEn 3240 kcal/kg, crude protein 14.95%, crude fiber 1.86% and 0.72% lysine; lactation AMEn 3510 kcal/kg, crude protein 19.92%, crude fiber 2.16% and 1.25% lysine). Feed was provided twice a day in the gestation phase and three times a day in the lactation phase. All animals (sows and piglets) had ad libitum water access with nipple drinkers.

### 2.3. Auditory Stimuli

Sows in treatments with auditory enrichment (MH-M and CH-M) were exposed to classical music every day, which was randomly chosen. Auditory enrichment was a 2 h playlist with Bach violin concertos with movements ranging from Largo (44 to 48 BPM) to Presto (180 to 200 BPM) tempos. The playlist was played three times a day, with 2 h intervals between each sequence; thus, the animals were exposed to a total of 6 h of music from 9 a.m. to 7 p.m.

The sound stimuli started 72 h after artificial insemination and lasted throughout the gestation and lactation phase of the sows. The auditory stimulus was reproduced via an amplified speaker (Frahm^®^ CM 600 BT, Frahm, Rio do Sul—SC, Brazil) with 200 W RMS power and USB input positioned in the shed so that all animals received a sound stimulus in which the intensity remained between 60 and 75 dB, measured with a decibel meter (AKSO^®^ AK823, Akso, São Leopoldo-RS, Brazil).

### 2.4. Body Surface and Eye Temperature

The surface and eye temperature of the sows were measured using an infrared thermography camera (FLIR thermal camera^®^ Teledyne Flir, Wilsonville—United States) throughout the experimental period once a week during gestation and twice a week during lactation. All measurements were taken on the same animals in the same phase throughout the experimental period. Images were captured at a distance of 1.0 m (perpendicular to the sow), at 7:30 a.m. and 11:30 a.m., prior to music reproduction, and at 10:30 a.m. and 2:30 p.m., during music reproduction.

**Figure 1:**
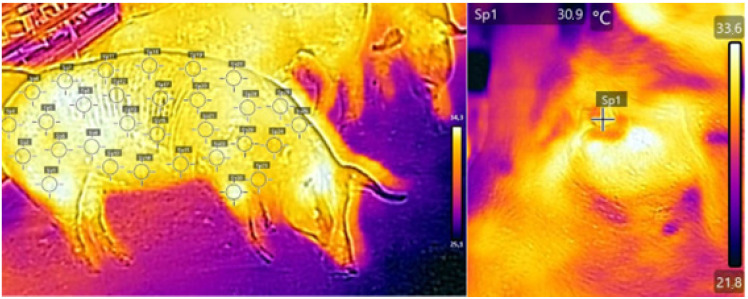
Thermographic image of the body of a sow with 30 points highlighted to calculate mean body surface temperature (**left**). Thermographic image of the eye of a sow with one highlighted point (**right**) during gestation.

Images were assessed using the specific software of the equipment (FLIR Report Studio^®^ Wilsonville—United States), where the reading of the color spectrum was converted into surface temperature. The coefficient of emissivity employed was 0.96 both for body surface and eye temperatures. The mean surface temperature and standard deviation of the body area were calculated using the temperature from 30 evenly distributed points selected to represent the overall body surface of the animals (Figure 1).

### 2.5. Reproductive Indices

The following reproductive indices of the sows were assessed: delivery duration (from the birth of the first piglet to placental expulsion), interval between births, number of live births, stillbirths, mummified piglets and individual piglet weight at birth and at weaning (21 days of age). Following the standard procedures of the farm for pig management, approximately 24 h after birth, litters were standardized in order to balance them in terms of weight and number of piglets per sow. This procedure was carried out between sows of the same treatment. After weaning the piglets, the weaning-estrus interval was evaluated, considering the number of days that elapsed between the weaning date and the 1st artificial insemination.

### 2.6. Statistical Analysis

The variables delivery duration, interval between births, live births, stillbirths, mummified piglets, piglet weight at birth and weaning, and surface and ocular temperatures (before and after the music playing) were evaluated for normality of the residues according to the Shapiro–Wilk test and for homogenicity of the variances according to Levene’s test.

Analysis of variance was performed using the PROC MIXED of the software [17], and data were analyzed as a randomized complete block design with block as a random effect. As there were sows with different parturition orders, this character was added to the mathematical model as a co-variable for data correction. The values on the surface and eye temperature were influenced by the ambient temperature at the time of measurement; thus, the ambient temperature was added to the mathematical model as a co-variable for data correction. The effects of interactions between the type of housing and auditory environmental enrichment were evaluated; when the interactions were significant, means were compared with Tukey’s test. When only principal effects were observed, means were compared using the F-test. All analyses used a 5% probability significance.

## 3. Results

### 3.1. Body Surface Temperature (ST) and Eye Temperature (ET) of Pregnant Sows

There was no interaction between the type of housing after insemination and environmental enrichment for ST or ET either before or during music reproduction.

However, a main effect of the type of housing and environmental enrichment on ET was observed in both periods evaluated (before exposure to music and during exposure to music). The ET of sows in collective housing was lower than those in the mixed housing system in both periods evaluated. Regarding auditory environmental enrichment, sows submitted to music stimuli had lower ET than those with no access to the auditory stimulus in both periods evaluated (Table 2).

No effect of housing was found on ST before or during music reproduction. Sows with auditory environmental enrichment exhibited higher ST compared to those in the control groups regardless of the type of housing assessed before music reproduction. However, during the reproduction of the auditory stimulus, no difference was seen among the groups (Table 2).

### 3.2. Body Surface Temperature (ST) and Eye Temperature (ET) of Lactating Sows

Before music reproduction, the ST of sows in the treatments with auditory environmental enrichment was lower than that in the control groups during the lactation period (Table 3).

An interaction was found between the type of housing and environmental enrichment only for ST during music reproduction. Sows from the collective housing (CH-C and CH-M) did not differ from each other; however, for sows from the mixed housing system (MH-C and MH-M), the surface temperature was higher for those submitted to the musical stimulus (MH-M). Comparing sows receiving auditory stimuli or not between housing systems (MH vs. CH), there was no difference in both comparisons (Table 3).

For eye temperature, there was only a main effect for the musical stimulus, sows exposed to music were lower than those with no access to the auditory stimulus during lactation both before and during music reproduction (Table 3).

### 3.3. Reproductive Indices

No interaction was observed between housing systems and environmental enrichment, nor any main effect of type of housing for any of the performance parameters assessed. No effect of auditory environmental enrichment was found on the weaning-to-estrus interval, delivery duration, number of live births, stillbirths, mummified piglets or weight at birth. However, piglets from sows submitted to auditory enrichment, regardless of the type of housing during gestation, were heavier at weaning than those in the control groups (Table 4).

## 4. Discussion

### Body Surface and Eye Temperature

During the gestation phase, the housing system after insemination had no effect on the sows surface temperature. However, sows in the CH groups exhibited lower eye temperature. In homeothermic animals, stress is related to an increase in body temperature and alteration in peripheric blood circulation [18,19,20]. Faraji and Metz [21] observed an increase in body surface temperature in rats submitted to space deprivation or contained. Foster and Ijichi [22] observed that cats living alone, with no others of the same species in the house, had higher eye temperatures than cats housed in groups. When an animal is stressed, the hypothalamus-hypophysis-adrenal axis is activated and produces heat due to an increase in catecholamine and cortisol concentrations, mediating physiological and behavioral alterations [20,23]. The central nervous system is primarily responsible for regulating body temperature; thus, brain temperature may be recognized as the core system temperature. Considering eye proximity with the brain, eye temperature is considered a good indicator of core temperature [24]. Blood flow in the eye is closely related to sympathetic activity [25]; stress responses may be detected using eye temperature variations. The present results indicated that the greater sensitivity of eye temperature in relation to skin temperature was an indicator of stress. Sows from mixed housing systems showed an increase in eye temperature independent of environmental enrichment; perhaps, skin temperature had no pronounced effects. Pigs are sensitive to high environmental temperatures, which may be explained by the keratinized sweat glands, in this sense, heat may strongly affect sows’ physiology [26]. However, in our study, the effect of environmental temperature was considered in the data analysis in an attempt to isolate its effect, allowing a possible more accurate assessment of the effect of stress on surface temperature, even so, no effect of treatments on surface temperature was observed, which reinforces the possibility of this parameter being less affected than the core temperature resulting from stress.

In the present study sows exposed to auditory enrichment exhibited lower eye temperature than those in the control. These findings were similar to Yáñez-Pizaña et al., [27] with piglets kept in environments enriched with ropes, bottles, flavorings and other toys and exhibited lower values of eye temperature compared to those kept in pens with no stimulus. A decrease in eye temperature may be related to relaxation provided by music, thus reducing ambiental stress [28]. Musical stimulus may lead to physical, mental and social alterations in individuals by activating brain regions responsible for cognitive and motor changes [29,30,31], particularly in the auditory association areas. Higher concentrations of neurotrophins in brain tissue have been associated with improved cognitive functions [30] and greater resilience against stress in studies that successfully used environmental enrichment [29].

No effect of gestation housing type on surface and eye temperatures of sows was observed during lactation, these findings may be related to the crates housing system during lactation, the same system for all the treatments. The effects of auditory enrichment on eye temperature of lactating sows were similar to those observed during gestation, suggesting music is a good tool for alleviating stress during the confinement period and also in individual farrowing crates.

## 5. Reproductive Indices

Regardless of the use of auditory environmental enrichment, no effects of type of housing during gestation were observed on sow reproductive indices, which indicates that the collective housing system may be considered a viable alternative to reduce the time of individual confinement without compromising economic and performance results. Gentilini et al. and Bampi et al., [32,33] found no differences in birth rates between the two housing systems, crates or collective pens. Mixed housing, in which sows are kept for about 28 to 35 days in individual crates after artificial insemination, mainly aims to avoid embryo losses, which is influenced by stress due to disputes to establish a social hierarchy or for food [9]; thus, fights related to social hierarchy may be observed in the first days of regrouping. Embryo implantation occurs between the 17th and 24th days after fertilization; thus, regrouping sows during that phase may increase embryonic mortality and, consequently, reduced fertilization rate or litter size [34]

According to Varley [35], regrouping should be done either before or after the embryo implantation period. If sows were regrouped 72 h after artificial insemination, the risk of embryonic resorption is reduced. This condition may explain the lack of difference in reproductive indices between types of housing.

However, it is important to highlight that fights between pregnant sows in collective housing do not occur only to establish social hierarchy and may last for longer periods, mainly in situations where sows were fed with a restricted diet at about 40 to 60% of their voluntary intake capacity, which results in a low degree of satiety and constant seeking for feed [36]. These stressful situations must also be under control in order to avoid embryonic losses.

There were no influences of auditory enrichment on most of the reproductive indices, except for piglet weight at weaning, which was higher for those from sows exposed to classical music during gestation and lactation. Fetal development is impacted by environmental factors. Thus, physical and psychological stressors in pregnant individuals impact lower offspring weight at birth, an increase in complications risks during delivery, greater incidence of abnormalities and neonatal mortality rates [37]. It was expected that exposing pregnant sows to music would be able to reduce stress and consequently increase the number of live births, weight at birth and decrease in embryonic mortality, which was not observed.

Auditory enrichment may help the development of innate behaviors, which benefits animal welfare and performance. The positive effects of music on piglets’ weaning weight may be related to the welfare of sows and piglets; stress reduces maternal care [38]. Lactating females in better conditions of welfare present reduced stress and stereotyped behaviors, resulting in a higher frequency of suckling. These facts may explain the better results of piglet weaning weight [38].

Additionally, piglets exposed to music had a lower expression of agonistic behaviors and an increased expression of playful behaviors [12,13,14,15,16,17,18,19,20,21,22,23,24,25,26,27,28,29,30,31,32,33,34,35,36,37,38,39]. The expression of abnormal behaviors, such as stereotypies and excessive aggressiveness, indicate unfavorable welfare conditions [40], which are related to facility issues and improper animal management, resulting in a decrease in productive performance. To emphasize the magnitude of these findings, considering the average difference in weaning weight observed in this study (0.746 kg), and estimating values of 2.4 births/sow/year, with an average of 13 weaned piglets per birth, auditory enrichment would be able to provide approximately 23,275 kg more of weaned piglets for every 1000 sows in the batch.

## 6. Conclusions

Collective or mixed housing systems do not affect sows’ reproductive performance, regardless of the use of auditory environmental enrichment. Exposing pregnant and lactating sows to classical music reduced their eye temperature, which suggests a decrease in stress inherent to these productive phases. Stress reduction resulted in higher piglet weight at weaning.

## Figures and Tables

**Table 1:** Treatments used in the research.

Description	Mixed Housing		Collective Housing	
	MH-C	MH-M	CH-C	CH-M
Duration of housing in cages after AI	35 days	35 days	72 h	72 h
Duration of housing in collective pens	72 days	72 days	104 days	104 days
Auditory environmental enrichment	NO	YES	NO	YES

MH-C = mixed housing with no music; MH-M = mixed housing with music; CH-C = collective housing with no music; CH-M = collective housing with music.

**Table 2:** Mean body surface temperature and eye temperature of pregnant sows in mixed housing or collective housing before and during exposure to auditory environmental enrichment.

Variable (%)	Music	Housing		Mean	SEM	*p*-Value		
		MH	CH			HOU	MUS	HOU * MUS
ST (°C)—BM	YES	34.75	34.69	34.68 A	0.027	0.1478	0.0184	0.3379
	NO	34.63	34.52	34.58 B				
	AVG	34.66	34.60	34.63				
ST (°C)—DM	YES	34.48	34.34	34.41	0.026	0.5065	0.8446	0.0571
	NO	34.36	34.45	34.41				
	AVG	34.42	34.40	34.41				
ET (°C)—BM	YES	33.23	33.16	33.19 B	0.037	0.0153	<0.0001	0.1341
	NO	33.93	33.66	33.79 A				
	AVG	33.58 a	33.41 b	33.52				
ET (°C)—DM	YES	33.41	33.31	33.36 B	0.035	0.0193	<0.0001	0.4437
	NO	34.41	34.22	34.32 A				
	AVG	33.91 a	33.77 b	33.89				

* Means followed by different capital letters in the same column and different small letters on the same row are statistically different according to Tukey’s test (*p* < 0.05). SEM = Standard error of the mean. HOU = Type of housing after AI. MUS = Music; ST = Mean body surface temperature; ET = eye temperature; MH = mixed housing; CH = collective housing; BM = before exposure to auditory environmental enrichment; DM = during exposure to auditory environmental enrichment.

**Table 3:** Mean body surface temperature and eye temperature of lactating sows in mixed housing or collective housing before and during exposure to auditory environmental enrichment.

Variable (%)	Music	Housing		Mean	SEM	*p*-Value		
		MH	CH			HOU	MUS	HOU * MUS
ST (°C)—BM	YES	34.97	34.84	34.90 B	0.046	0.5197	0.0361	0.2285
	NO	35.05	35.09	35.07 A				
	AVG	35.01	34.96	34.990				
ST (°C)—DM	YES	34.9 Aa	34.65 Aa	34.77	0.048	0.4407	0.2402	0.0211
	NO	34.61 Ba	34.74 Aa	34.68				
	AVG	34.75	34.69	34.72				
ET (°C)—BM	YES	34.47	34.37	34.42 B	0.038	0.2761	0.0226	0.9231
	NO	34.64	34.56	34.6 A				
	AVG	34.55	34.47	34.52				
ET (°C)—DM	YES	34.23	34.20	34.21 B	0.031	0.8351	<0.0001	0.6812
	NO	34.83	34.84	34.83 A				
	AVG	34.53	34.52	34.55				

* Means followed by different capital letters in the same column and different small letters on the same row are statistically different according to Tukey’s test (*p* < 0.05). SEM = Standard error of the mean. HOU = Type of housing after AI. MUS = Music; ST = Mean body surface temperature; ET = eye temperature; MH = mixed housing; CH = collective housing; BM = before exposure to auditory environmental enrichment; DM = during exposure to auditory environmental enrichment.

**Table 4:** Reproductive indices of sows from mixed (MH) or collective (CH) housing exposed or not to auditory environmental enrichment.

Variable	Music	Housing		Mean	SEM	*p*-Value		
		MH	CH			HOU	MUS	HOU * MUS
Weaning-to-estrus interval (days)	YES	5.31	4.50	4.9	0.307	0.5884	0.077	0.4335
	NO	3.73	3.88	3.81				
	AVG	4.54	4.18	4.35				
Birth Weight (kg)	YES	1.379	1.328	1.353	0.011	0.3016	0.204	0.1486
	NO	1.313	1.323	1.318				
	AVG	1.346	1.325	1.335				
Weaning Weight (kg)	YES	6.294	6.346	6.320 A	0.047	0.2778	<0.0001	0.6021
	NO	5.500	5.648	5.574 B				
	AVG	5.897	5.997	5.927				
Livebirths (n)	YES	18.166	16.071	17.119	0.484	0.0847	0.35	0.6798
	NO	16.857	15.562	16.209				
	AVG	17.511	15.817	16.571				
Stillbirths (n)	YES	0.666	0.428	0.538	0.097	-	-	-
	NO	0.857	0.500	0.666				
	AVG	0.769	0.466	0.607				
Mummified (n)	YES	0.583	0.357	0.461	0.104	-	-	-
	NO	0.285	0.625	0.466				
	AVG	0.423	0.500	0.464				
Delivery duration (min)	YES	276	212	244	12.467	0.1849	0.731	0.2374
	NO	237	234	235				
	AVG	257	223	239				

* Means followed by different capital letters in the same column statistically differed according to Tukey’s test (*p* < 0.05). SEM = Standard error of the mean. HOU = Type of housing after AI. MUS = Music. - Due to the low occurrence of mummified and stillbirths, it was not possible to statistically evaluate the data. Only observed averages are presented.

## Data Availability

The data that support this study will be shared upon reasonable request to the corresponding author.
